# The cyclohexene derivative MC-3129 exhibits antileukemic activity via RhoA/ROCK1/PTEN/PI3K/Akt pathway-mediated mitochondrial translocation of cofilin

**DOI:** 10.1038/s41419-018-0689-4

**Published:** 2018-05-29

**Authors:** Yi Zheng, Qin Ouyang, Ruoqiu Fu, Lei Liu, Hongwei Zhang, Xiaoye Hu, Yanxia Liu, Yingchun Chen, Ning Gao

**Affiliations:** College of Pharmacy, Third Military Medical University, 400038 Chongqing, China

## Abstract

The effects of MC-3129, a synthetic cyclohexene derivative, on cell viability and apoptosis have been investigated in human leukemia cells. Exposure of leukemia cells to MC-3129 led to the inhibition of cell viability and induction of apoptosis through the dephosphorylation and mitochondrial translocation of cofilin. A mechanistic study revealed that interruption of the RhoA/ROCK1/PTEN/PI3K/Akt signaling pathway plays a crucial role in the MC-3129-mediated dephosphorylation and mitochondrial translocation of cofilin and induction of apoptosis. Our in vivo study also showed that the MC-3129-mediated inhibition of the tumor growth in a mouse leukemia xenograft model is associated with the interruption of ROCK1/PTEN/PI3K/Akt signaling and apoptosis. Molecular docking suggested that MC-3129 might activate the RhoA/ROCK1 pathway by targeting LPAR2. Collectively, these findings suggest a hierarchical model, in which the induction of apoptosis by MC-3129 primarily results from the activation of RhoA/ROCK1/PTEN and inactivation of PI3K/Akt, leading to the dephosphorylation and mitochondrial translocation of cofilin, and culminating in cytochrome c release, caspase activation, and apoptosis. Our study reveals a novel role for RhoA/ROCK1/PTEN/PI3K/Akt signaling in the regulation of mitochondrial translocation of cofilin and apoptosis and suggests MC-3129 as a potential drug for the treatment of human leukemia.

## Introduction

Chemotherapy is one of the most important treatments for cancer. A principal obstacle to the clinical efficacy of chemotherapy is the potential toxicity to normal tissues of the body and the development of drug resistance^1,2^. To overcome these obstacles, the design and discovery of efficient and safe chemical agents for the treatment of cancer is the primary objective of contemporary medicinal chemistry. Recently, asymmetric organocatalysis has been successfully applied to synthesize new chemical derivatives based on the bioactivity and mechanism of anticancer activity^3^. In recent years, cyclohexene derivatives containing chiral primary amines have received considerable attention because of their diverse chemotherapeutic potential, including versatile anticancer activities. Previous studies have reported that 2-cyclopentenones and 2-cyclohexenone in the presence of chiral primary amines exhibited promising activity against some cancer cell lines, thus indicating that such skeletons might serve as leads in drug discovery^4^. Synthetic tricyclic pyranopyrones with simple aromatic substituents possess anticancer properties^5^. Compound ZJ-101, containing a cyclohexenyl ring, also exhibited anticancer activity^6^. ST7612AA1, a new generation of HDAC inhibitors, exhibited potential activity against a broad panel of cancer cell lines and in vivo tumor models^7^. These reports indicated that synthetic cyclohexene derivatives might show potential as chemotherapeutic agents for the treatment of human cancer.

A specific pharmacological mechanism is important for further drug development. Increasing evidence has revealed that the high incidence of Rho-associated coiled-coil-containing protein kinase 1 (ROCK1) overexpression in human tumors suggests that this kinase is important in the carcinogenic process and therefore is a potential target for therapeutic intervention^8^. ROCK1 belongs to a family of serine/hreonine kinases activated by Rho GTPases or caspase-3 via cleavage of the C-terminal auto-inhibitory domain from the kinase active site^9^. ROCK1 is of significant interest in drug discovery, owing to its fundamental role in vital signal transduction pathways central to many essential cellular activities, including cell death and survival^10^. Several ROCK1 inhibitors are involved in the regulation of cell death and survival through distinct mechanisms (i.e., Mcl-1 phosphorylation and PTEN/PI3K/Akt signaling pathway)^11,12^.

In addition to biological studies, computer modeling is an important tool for target identification, since the 3D structures of certain targeted receptor(s) could be constructed by homological modeling, and the binding poses and binding affinities of the ligand and receptor could be predicted by molecular docking^13^. Homology modeling refers to constructing an atomic-resolution model of the “target” protein from its amino acid sequence and an experimental three-dimensional structure of a related homologous protein. Docking involves fitting virtual ligands, typically derived from large virtual libraries, into targeted binding sites employing computer algorithms. A well-studied virtual ligand–protein interaction complex model is an essential prerequisite toward the design and subsequent optimization of novel bioactive compounds, including new anticancer agents^14^.

In a recent study, we reported nine new cyclohexene derivatives, synthesized through exo-Diels–Alder and redox reactions, which exhibited a different degree of anticancer activities^4^. Here, we found a new cyclohexene derivatives, named MC-3129, also exhibited potent cytotoxic effects against several human cancer cell lines. The molecular mechanism of MC-3129-mediated apoptosis involves RhoA/ROCK1/PTEN activation and PI3K/Akt inactivation, leading to the dephosphorylation and mitochondrial translocation of cofilin. MC-3129 was also evaluated in vivo for its antileukemic activity in a U937 xenograft mouse model. Molecular docking further confirmed that MC-3129 was precisely docked into the binding pocket of LAPR2 with an obvious hydrogen bond between LAPR2 and MC-3129, suggesting that MC-3129 might activate the RhoA/ROCK1 pathway by targeting LPAR2. The ability of MC-3129 to induce apoptosis in leukemia cells and solid tumor cells makes this chemical a promising candidate for the development of a relapse-free therapeutic regimen for leukemia and potentially other types of cancer.

## Results

### MC-3129 reduces cell viability in leukemia cells and other cancer cell lines

The chemical library was assembled with processing series of synthetic strategies on asymmetric organocatalysis over a number of years. Nearly, 3000 small molecules were screened for their anti-proliferative activity on cancer cells. Among these molecules, MC-3129, a cyclohexene derivative synthesized though exo-Diels–Alder and redox reactions, exhibited the most potent cytotoxic effects against human leukemia U937 cells (Table 1 and Figure S1). In addition to the U937 cell line, other three leukemia cell lines (Jurkat, HL-60, and K562) and five solid tumor-derived cell lines, including A549 (non-small cell lung cancer), SMMC-7721 (hepatocellular carcinoma), Eca109 (esophageal carcinoma), DU145 (prostate carcinoma), and MDA-MB-231 (breast adenocarcinoma), were tested for the cytotoxic effects of MC-3129. As shown in Table 1 and Figure S1, MC-3129 exhibited inhibitory effects on cell viability in a dose-dependent manner in these cancer cell lines.

**Table 1:** Cellular evaluation of the cyclohexene derivative against diverse cancer cell lines


Cyclohexene derivative	Cell lines	IC_50_ (μM, means ± SD)
MC-3134	U937	37.65 ± 4.81
MC-3135	U937	88.52 ± 13.31
	U937	10.17 ± 0.34
	Jurkat	15.30 ± 3.27
	HL-60	15.95 ± 1.90
MC-3129	K562	17.04 ± 2.34
	MDA-MB-231	13.41 ± 2.23
	DU145	16.99 ± 2.35
	A549	30.13 ± 2.52
	Eca109	20.50 ± 2.55
	SMMC-7721	23.57 ± 2.74

### MC-3129 induces apoptosis in human leukemia cells

**Fig. 1:**
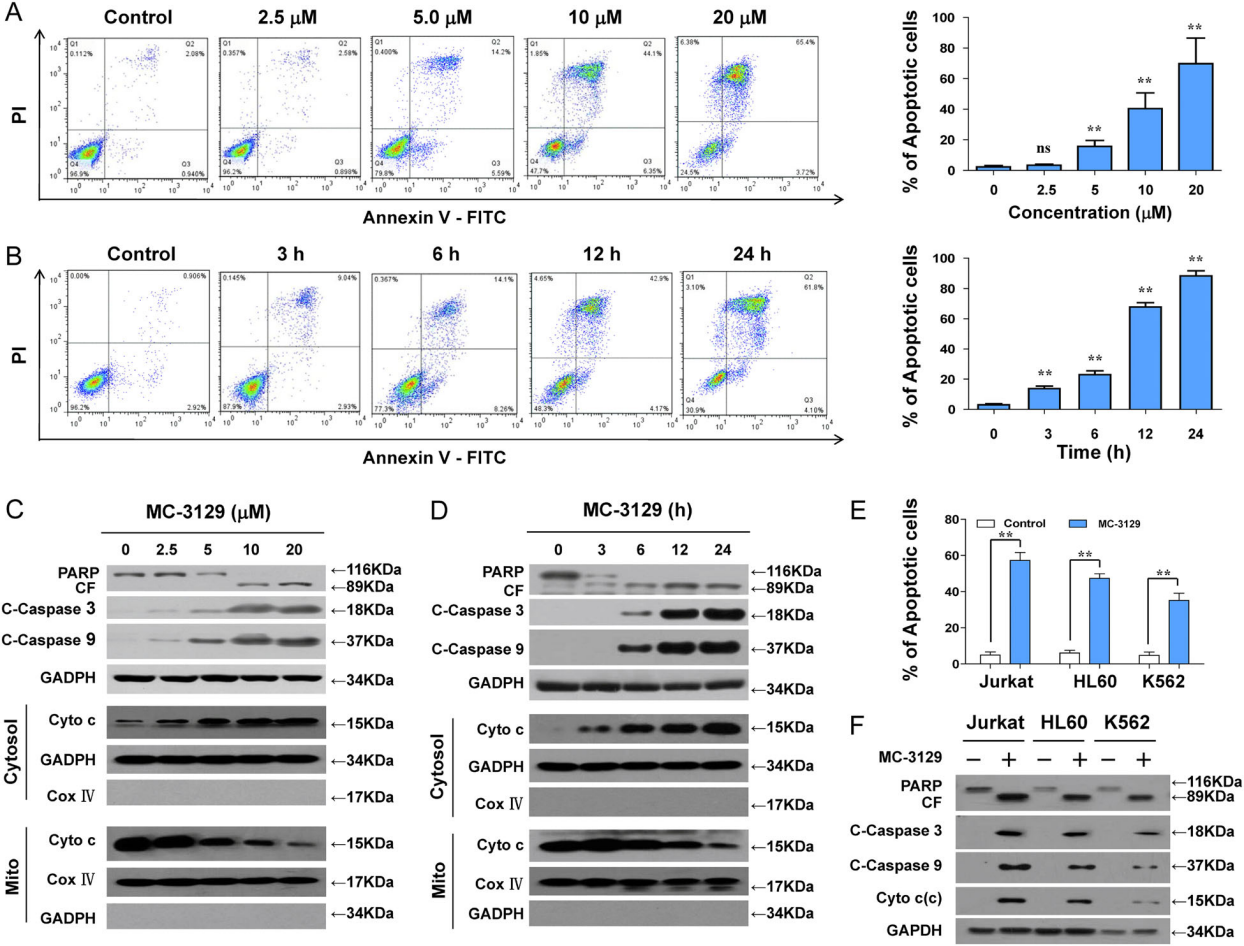
MC-3129 induces apoptosis in multiple leukemia cell lines. U937 cells were treated with various MC-3129 concentrations for 24 h or with 10 μM of MC-3129 for different time intervals, as indicated. **a**, **b** The cell apoptosis was determined by flow cytometry using Annexin V/PI staining, the percentage of apoptotic cells was analyzed for three separate experiments (mean ± SD, ns *P* > 0.05, **P *<* 0.01 compared with control). **c**, **d** Whole cell lysates, cytosolic (Cytosol) and mitochondrial (Mito) fractions from U937 cells were prepared and subjected to immunoblotting using antibodies against PARP, cleaved-caspase-3 (C-Caspase-3), cleaved-caspase-9 (C-Caspase 9), cytochrome c (Cyto c), GADPH, and Cox IV. Jurkat, HL-60, and K562 cells were treated with or without 10 μM of MC-3129 for 24 h. **e** The cell apoptosis was determined by flow cytometry, values represent the means ± SD, for three separate experiments (**P < 0.01). **f** The total protein lysates were analyzed by immunoblotting using the indicated antibodies. CF cleavage fragment

We next examined the effects of MC-3129 on apoptosis in human leukemia cell lines. Flow cytometry analysis revealed that the exposure of U937 cells to MC-3129 resulted in a significant increase in apoptosis in dose- and time-dependent manners (Fig. 1a, b). Consistent with these findings, the same MC-3129 concentrations and exposure intervals caused the cleavage/activation of caspase-9 and caspase-3 and degradation of PARP. These events were also accompanied by significant increases in the release of cytochrome c from mitochondria into the cytosol (Fig. 1c, d).

To determine whether these events were restricted to myeloid leukemia cells, parallel studies were performed in Jurkat, HL-60, and K562 leukemia cell lines. These cells exhibited the apoptotic effects of MC-3129 similar to those observed in U937 cells (Fig. 1e). Additionally, Jurkat, HL-60, and K562 cells exhibited comparable degrees of caspase-9 and caspase-3 activation, PARP degradation, as well as cytochrome c release (Fig. 1f).

### Exposure of MC-3129 results in the downregulation of Mcl-1, dephosphorylation of Bad, and mitochondrial translocation of Bax

**Fig. 2:**
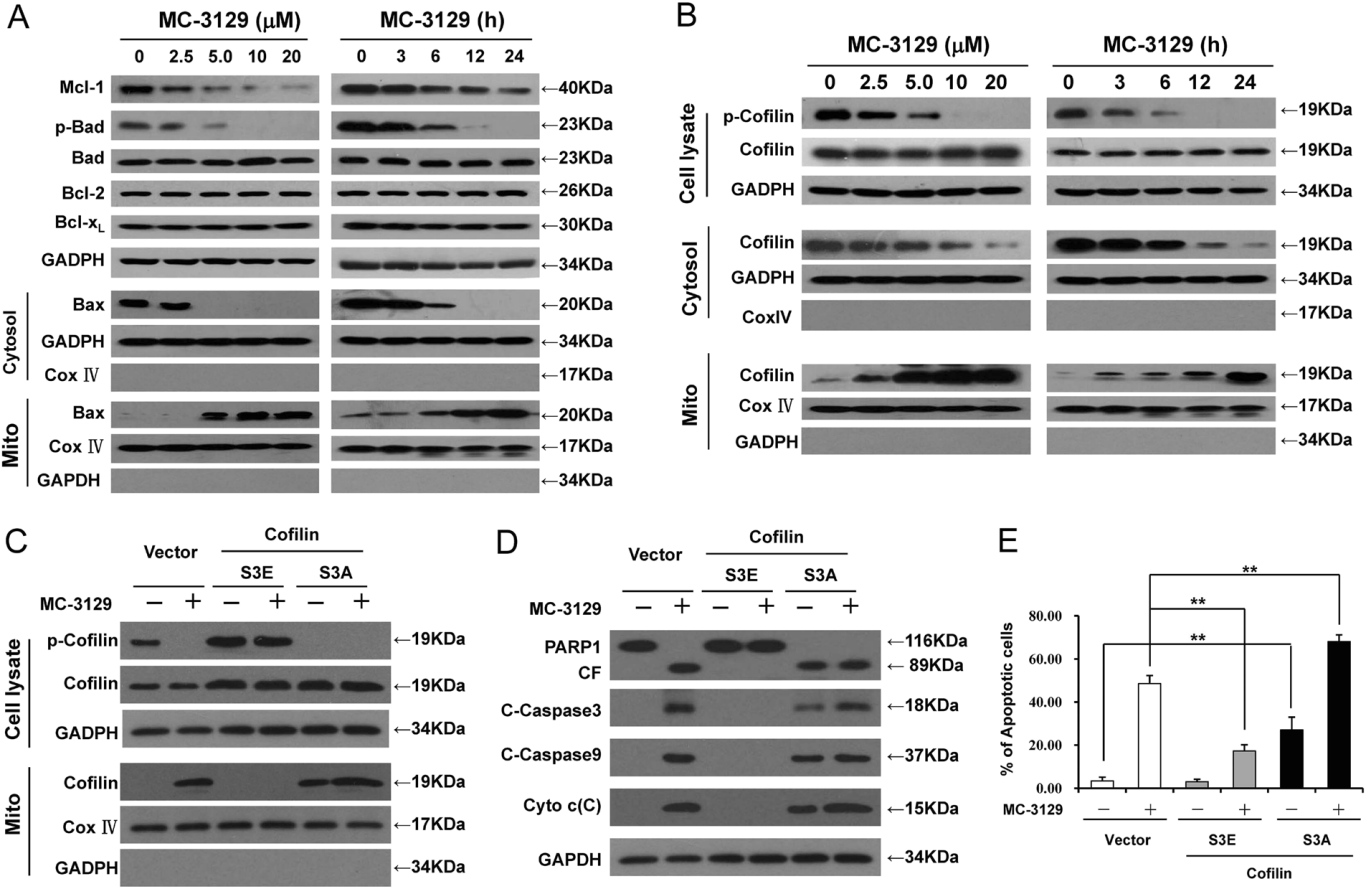
Exposure of MC-3129 results in the dephosphorylation and mitochondrial translocation of cofilin. U937 cells were treated with various MC-3129 concentrations for 24 h or with 10 μM of MC-3129 for different time intervals, as indicated. **a** Whole cell lysates were prepared and subjected to western blot analysis using antibodies against Mcl-1, phospho-Bad (p-Bad), Bad, Bcl-2, Bcl-xL, and GADPH. The cytosolic (Cytosol) and mitochondrial (Mito) fractions were also prepared and subjected to western blot analysis using antibodies against Bax, GADPH, and Cox IV. **b** Whole cell lysates, cytosolic (Cytosol), and mitochondrial (Mito) fractions were analyzed by western blot assay using antibodies against phospho-cofilin (p-cofilin), cofilin, GADPH, and Cox IV. U937 cells were transfected with control empty vectors or pseudophosphorylated (inactive, S3E) mutant plasmids or human cofilin dephosphorylated (active, S3A) mutant plasmids for 48 h, and then were treatment with 10 μM MC-3129 for 24 h. **c**, **d** Whole cell lysates and mitochondrial fractions were determined by immunoblotting. **e** The cell apoptosis was determined by flow cytometry using Annexin V/PI staining, the percentage of apoptotic cells was analyzed for three separate experiments (mean ± SD, **P < 0.01)

Since B cell lymphoma-2 (Bcl-2) family proteins are key regulators of apoptosis^15^, we next examined the dose- and time-dependent effects of MC-3129 on the expression of various Bcl-2 family members. As shown in Fig. 2a, the exposure of U937 cells to MC-3129 resulted in decreases in the levels of Mcl-1 (myeloid cell leukemia 1) and phospho-Bad (Bcl-2-associated death promoter) in a dose- and time-dependent manner. In contrast, treatment with MC-3129 did not discernibly modify the expression of other Bcl-2 family proteins, including Bcl-2 and Bcl-xL (B cell lymphoma-extra large). However, treatment with MC-3129 resulted in a pronounced redistribution of Bax from the cytosol to the mitochondria, a hallmark of apoptosis induction via the mitochondrial pathway.

### Exposure of MC-3129 results in the dephosphorylation and mitochondrial translocation of cofilin

Recent evidence has indicated that the dephosphorylation/mitochondrial translocation of cofilin is crucial for the initiation of mitochondrial injury-mediated apoptosis^16,17^. We next investigated whether MC-3129 could affect the dephosphorylation/mitochondrial translocation of cofilin during the initiation of apoptosis. Treating cells with MC-3129 decreased the levels of phospho-cofilin (Ser3) in whole cell lysate, increased the levels of cofilin in mitochondria and decreased the levels of cofilin in the cytosol in dose- and time-dependent manners (Fig. 2b). Similar results were also obtained in other cancer cell lines (Figure S2A). To futher determine whether the phosphorylation status of cofilin could influence its ability to translocate to mitochondria and induce apoptosis mediated by MC-3129, two cofilin mutants were generated that mimick either the dephosphorylated or phosphorylated forms by changing Ser3 to alanine (active; S3A) or glutamic acid (inactive; S3E) as described previously^17^. Overexpression of cofilin S3A enhanced, whereas cofilin S3E abolished, the mitochondrial translocation localization of cofilin (Fig. 2c). Furthermore, cofilin S3A enhanced, whereas cofilin S3E reduced cytochrome c release, caspase-3 activation and apoptosis mediated by MC-3129 (Fig. 2d, e). Thus, these findings suggest that MC-3129-mediated dephosphorylation of cofilin (Ser3) is required for the mitochondrial translocation of cofilin and induction of apoptosis.

### Exposure of MC-3129 activates RhoA/ROCK1/PTEN and inactivates of PI3K/Akt

**Fig. 3:**
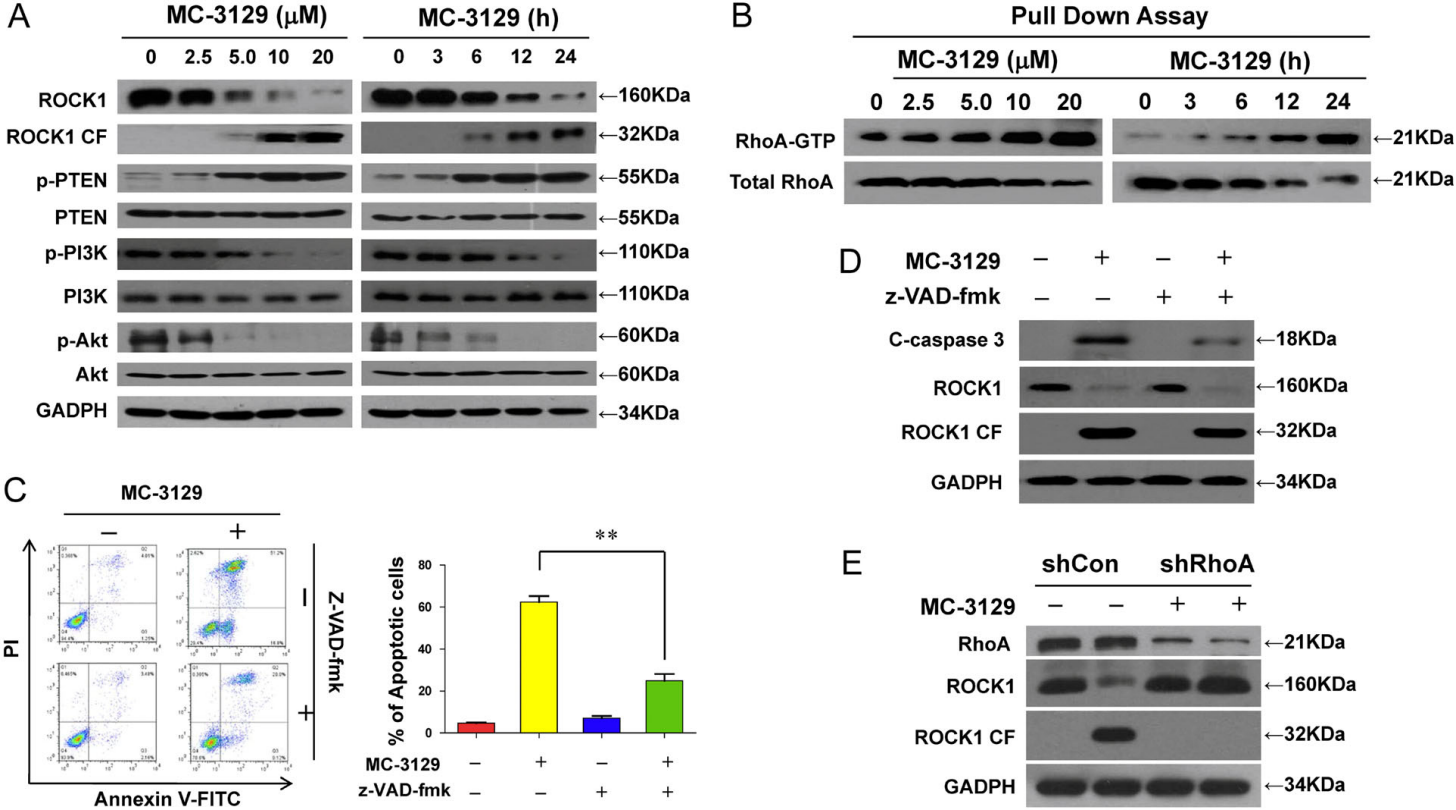
Exposure of MC-3129 activates RhoA/ROCK1/PTEN and inactivates of PI3K/Akt. **a** U937 cells were treated with various MC-3129 concentrations for 24 h or with 10 μM of MC-3129 for different time intervals, as indicated. Whole cell lysates were prepared and subjected to western blot analysis using antibodies against against ROCK1, phospho-PTEN (p-PTEN), PTEN, phospho-PI3K (p-PI3K), PI3K, phospho-Akt (Ser473, p-Akt), Akt, and GADPH. **b** Active RhoA-GTP was pulled down by glutathione S-transferase linked to the RhoA-binding domain of Rhotekin (GST-RBD); bead/protein complexes and total RhoA were detected by immunoblotting with anti-RhoA. U937 cells were pretreated with the caspase inhibitor Z-VAD-fmk (20 μM) for 2 h, followed by treatment with 10 μM of MC-3129 for 24 h. **c** Apoptosis was determined by flow cytometry using Annexin V/PI staining, the percentage of apoptotic cells was analyzed for three separate experiments (mean ± SD, **P < 0.01). **d** Total protein lysates were analyzed by immunoblotting using antibodies against cleaved-Caspase 3 (C-Caspase 3), ROCK1, cleaved-ROCK1 (ROCK1 CF), and GAPDH. **e** U937 cells were stably transfected with lentivirus containing RhoA-siRNA (shRhoA) or a scrambled control siRNA (shCon), cells were treated with or without 10 μM of MC-3129 for 24 h. Total protein lysates were analyzed by immunoblotting using antibodies against RhoA, ROCK1, cleaved-ROCK1 (ROCK1 CF), and GAPDH

The effects of MC-3129 on U937 cells were examined in relation to changes in various signal transduction pathways implicated in the regulation of apoptosis. The exposure of U937 cells to MC-3129 decreased ROCK1 levels and increased ROCK1 cleavage in dose- and time-dependent manners (Fig. 3a). Similar results were also obtained in other cancer cell lines (Figure S2B). Treating cells with MC-3129 also resulted in discernable increases in the levels of phospho-PTEN and decreases in the levels of phospho-PI3K and phospho-Akt in dose- and time-dependent manners (Fig. 3a). Similar results were also obtained in other cancer cell lines (Figure S2B). Furthermore, MC-3129 exposure induced dose- and time-dependent increases in the GTP-activated form of RhoA (Fig. 3b).

Because perturbations of ROCK1 activation in MC-3129-treated cells could represent secondary events stemming from caspase activation, parallel studies were performed in cells exposed to the broad caspase inhibitor z-VAD-fmk. As anticipated, pretreatment with z-VAD-fmk effectively blocked MC-3129-induced apoptosis (Fig. 3c) and caspase-3 cleavage/activation (Fig. 3d). However, pretreatment with z-VAD-fmk did not prevent MC-3129**-**mediated ROCK1 activation (Fig. 3d). To confirm whether RhoA could activate ROCK1 during MC-3129-induced apoptosis, a lentivirus shRNA approach was used to stably knockdown RhoA expression. Depletion of RhoA with shRNA attenuated MC-3129-mediated ROCK1 activation (Fig. 3e). Such findings indicate that MC-3129-mediated perturbations in RhoA/ROCK1/PTEN/PI3K/Akt signaling and apoptotic regulatory events proceed through a caspase-independent pathway.

### Inhibition or knockdown of ROCK1 abrogates the MC-3129-mediated dephosphorylation and mitochondrial translocation of cofilin and induction of apoptosis

**Fig. 4:**
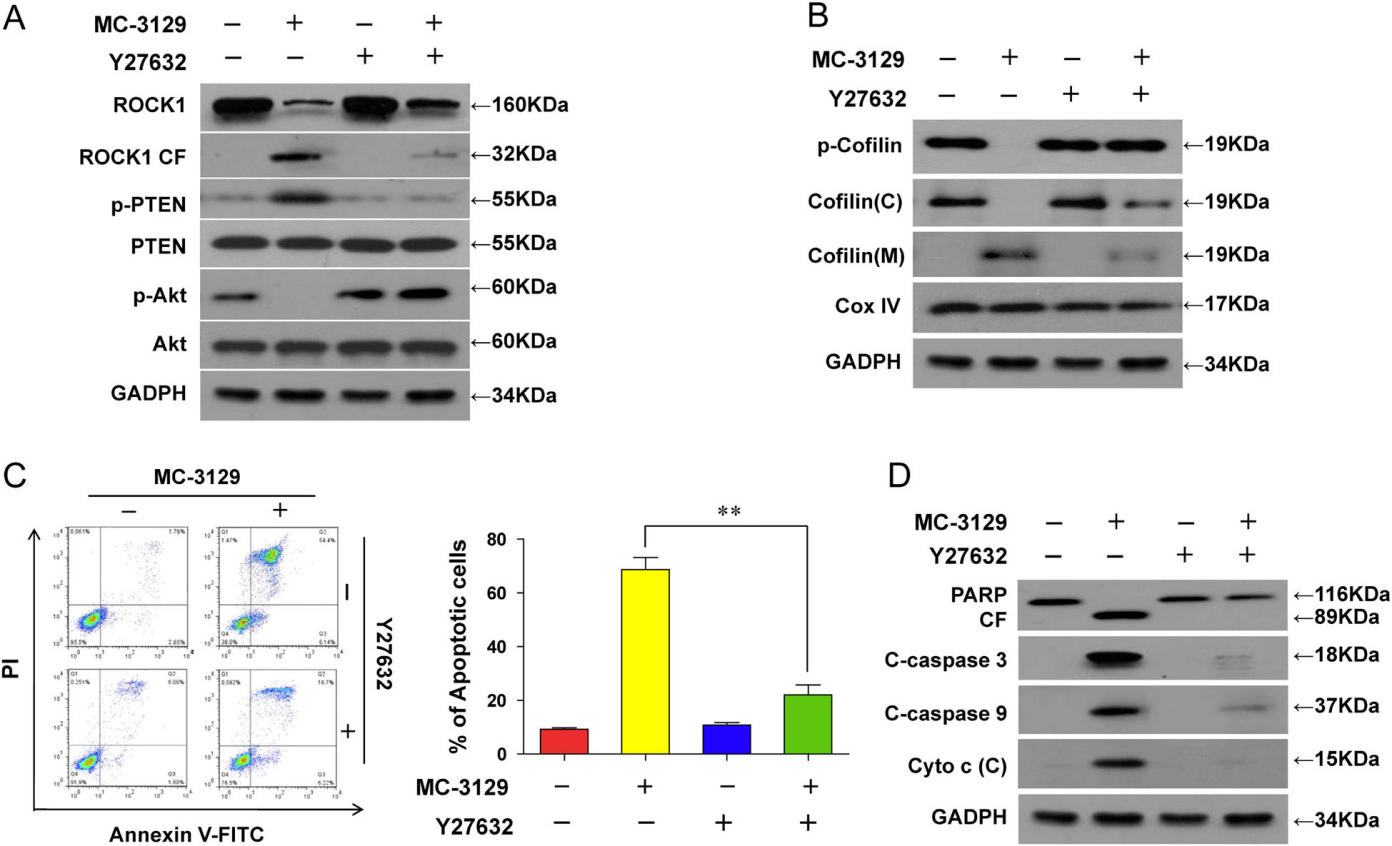
Inhibition of ROCK1 activity by Y27632 attenuated MC-3129-induced apoptosis in U937 cells. U937 cells were pretreated with 20 μM of Y27632, a ROCK1 inhibitor, for 2 h, followed by treating with 10 μM of MC-3129 for 24 h. **a**, **b** Total cellular extracts, cytosolic (C) and mitochondrial (M) fractions were prepared and subjected to western blot analysis using antibodies against ROCK1, p-PTEN, PTEN, p-Akt, Akt, p-cofilin, cofilin, GADPH, and Cox IV. **c**, **d** Apoptosis was determined by flow cytometry with Annexin V/PI staining and total protein lysates were analyzed by immunoblotting using the indicated antibodies. Error bars represent the means ± SD for three separate experiments. **P *<* 0.01

To further assess the functional significance of ROCK1 activation in regulating the dephosphorylation and mitochondrial translocation of cofilin and induction of apoptosis, a ROCK1 inhibitor Y27632 was employed. Pretreating cells with Y27632 decreased MC-3129-mediated ROCK1 cleavage/activation, PTEN activation, and Akt inactivation (Fig. 4a). Pretreatment with Y27632 also decreased the MC-3129-mediated dephosphorylation and mitochondrial translocation of cofilin (Fig. 4b). Furthermore, pretreatment with Y27632 attenuated MC-3129-mediated apoptosis (Fig. 4c), accompanied by decreases in the degradation of PARP, cleavage/activation of caspase-9 and caspase-3, as well as the release of cytochrome c into the cytosol (Fig. 4d).

**Fig. 5:**
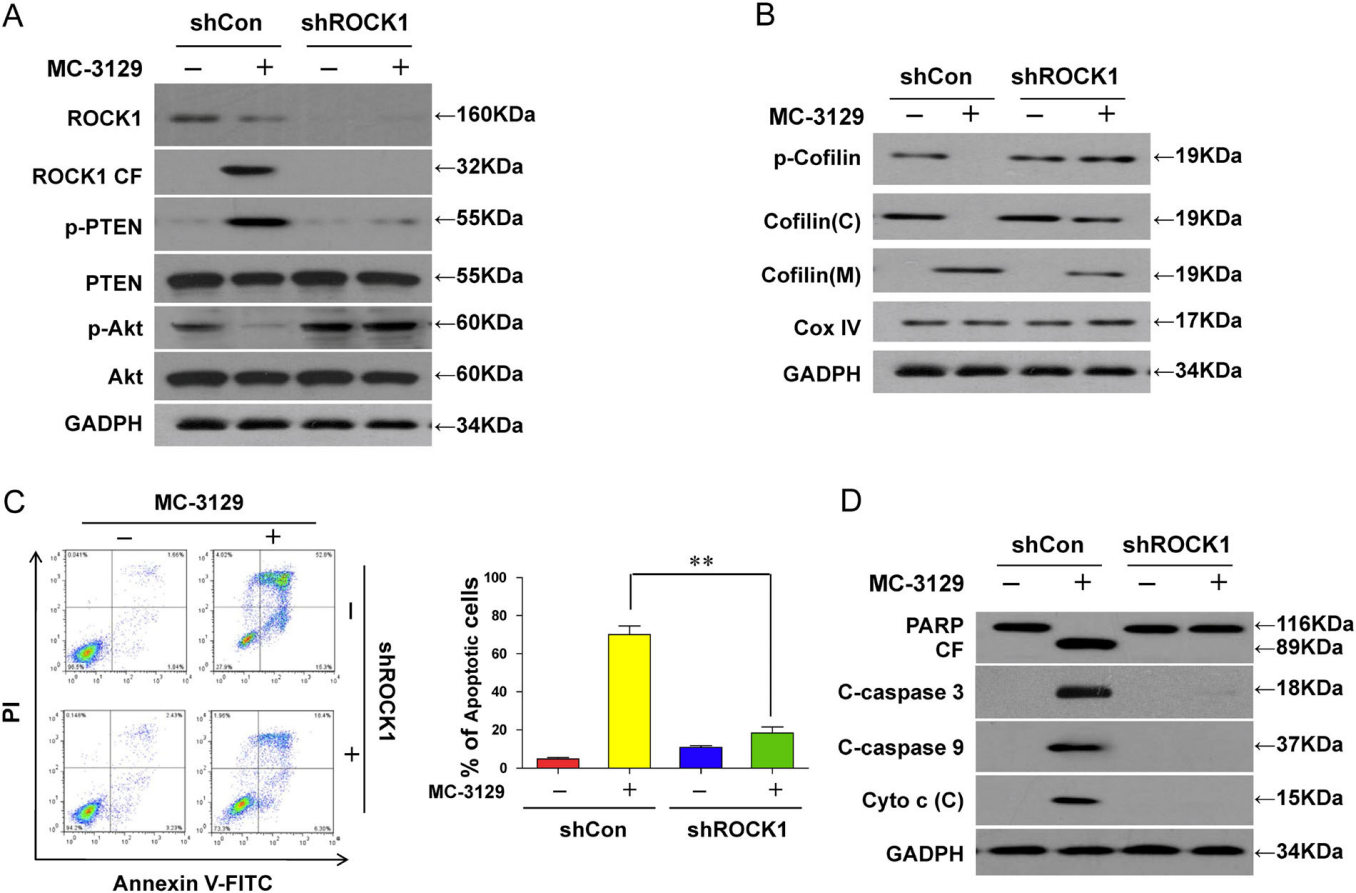
Inhibiting ROCK1 activation attenuated MC-3129-induced apoptosis. U937 cells were stably transfected with lentivirus containing ROCK1-specific siRNA (shROCK1) or a scrambled control siRNA (shCon), cells were treated with or without 10 μM of MC-3129 for 24 h. **a**, **b** Total cellular extracts, cytosolic (C) and mitochondrial (M) fractions were prepared and subjected to western blot analysis using anti-ROCK1, p-PTEN, PTEN, p-Akt, Akt, p-cofilin, cofilin, GADPH, and Cox IV. **c**, **d** Apoptosis was measured by flow cytometry using Annexin V/PI staining, and total protein lysates were analyzed by immunoblotting using the indicated antibodies. Error bars represent the means ± SD for three separate experiments. **P *<* 0.01

To further confirm the functional role of ROCK1 in MC-3129-mediated dephosphorylation and mitochondrial translocation of cofilin and apoptosis, a lentivirus shRNA approach was used to stably knockdown ROCK1 expression. The infection of U937 cells with ROCK1 shRNA reduced the expression of ROCK1 and resulted in blockage in MC-3129-mediated ROCK1 cleavage/activation, PTEN activation and Akt inactivation (Fig. 5a). The knockdown of ROCK1 also attenuated the MC-3129-mediated dephosphorylation and mitochondrial translocation of cofilin (Fig. 5b). In addition, the knockdown of ROCK1 attenuated MC-3129-mediated apoptosis (Fig. 5c), degradation of PARP, cleavage/activation of caspase-9 and caspase-3, as well as the release of cytochrome c into the cytosol (Fig. 5d). Taken together, these findings indicate that activation of ROCK1 played an important functional role in the MC-3129-mediated dephosphorylation and mitochondrial translocation of cofilin and apoptosis.

### MC-3129 inhibits tumor growth in a U937 xenograft mouse model

**Fig. 6:**
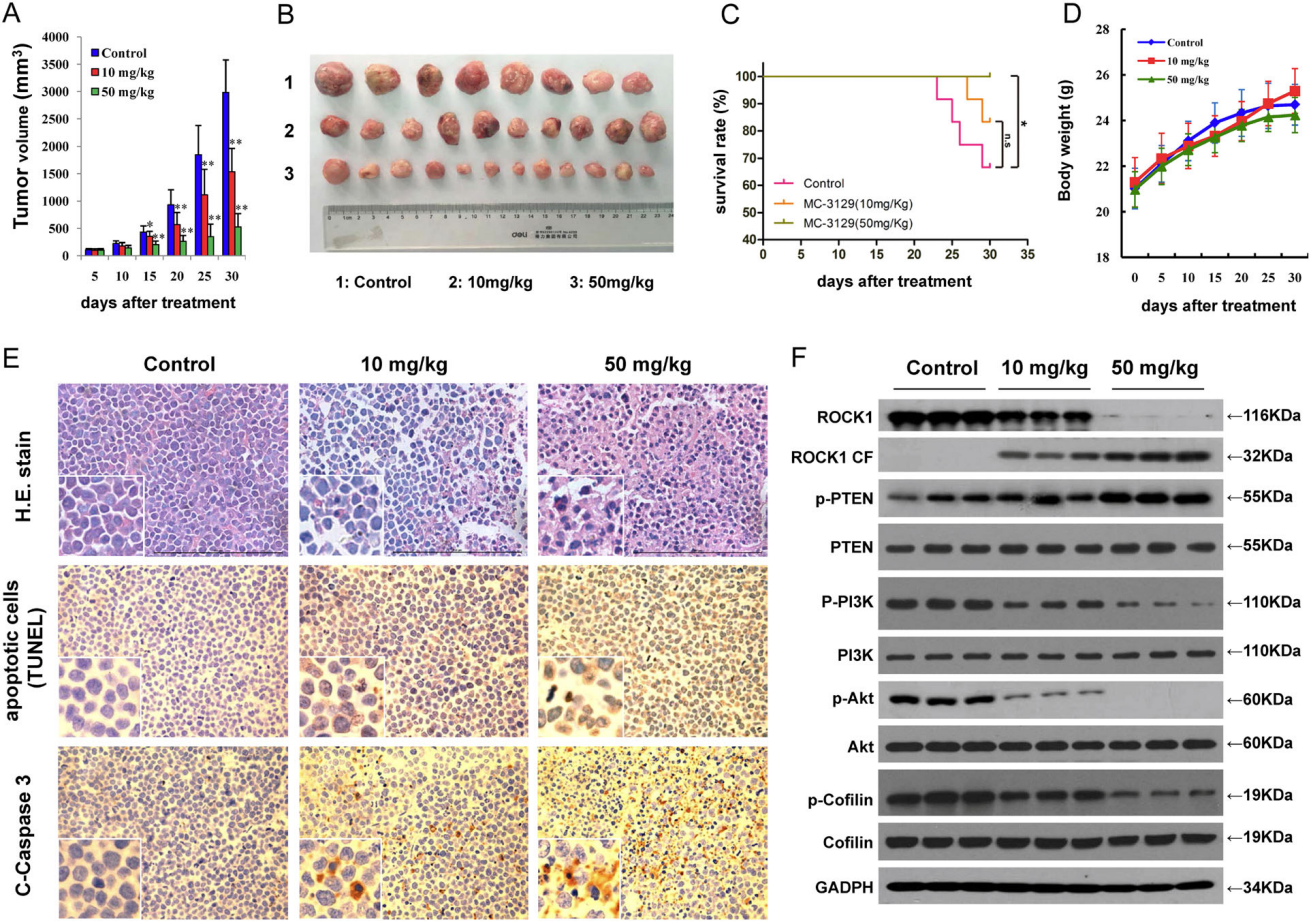
MC-3129 inhibits tumor growth and induces apoptosis in a U937 xenograft animal model. Thirty-six nude mice (5 weeks old) were inoculated subcutaneously with U937 cells (2 × 10^6^ cells/mouse) and randomly divided into three groups (12/group) for treatment with MC-3129 (10 mg/kg, 50 mg/kg, i.p., five times per week) or with vehicle control solvent as described in the “Methods” section. Tumor growth was measured once every 5 days, and tumor volume (V) was calculated as *V* = *lw*^2^/2. **a**, **b** Average tumor volume and gross appearance in the vehicle control group and the MC-3129 treatment groups. Error bars represent the means ± SD. *P < 0.05 or **P < 0.01 compared with control. **c** The Kaplan–Meier survival curve of the control group and the MC-3129 treatment groups during the 30 days of treatment. ^ns^*P* > 0.05, **P < 0.01. **d** Body weight changes of mice during the 30 days of study. Statistical analysis of body weight changes showed no significant differences between the MC-3129 treatment and vehicle control groups. **e** Tumor tissues were sectioned and subjected to H&E staining, TUNEL assay, and immunohistochemistry for evaluating histological morphology, apoptosis, and expression of C-Caspase-3. **f** Whole cell lysates were prepared and subjected to western blot analysis using antibodies against ROCK1, p-PTEN, PTEN, p-PI3K, PI3K, p-Akt, Akt, p-Cofilin, Cofilin, and GADPH

To determine whether our in vitro findings could be replicated in vivo, a U937 cell xenograft tumor growth model was employed. Nude mice were subcutaneously inoculated with U937 cells, followed by injections with vehicle or MC-3129 (10 and 50 mg/kg, i.p.) for 30 days starting 3 days after tumor inoculation. As shown in Fig. 6a, b, treatment of nude mice with MC-3129 resulted in a significant suppression of tumor growth after 15 days of drug exposure. The average tumor volumes for the 10 and 50 mg/kg MC-3129 treatment groups were 356.2 ± 88.9 mm^3^ and 205.5 ± 64.2 mm^3^, respectively, compared to that for the vehicle control group (433.5 ± 110.6 mm^3^) (*P* *<* 0.05 or *P* *<* 0.01). These effects became more apparent after 20 days of drug exposure. At 30 days after drug exposure, the average tumor volumes were strongly reduced by ~18% and 50% at two concentration levels (10 and 50 mg/kg, respectively) compared with that of the vehicle control group (*P* *<* 0.01). The results of Kaplan–Meier survival analysis showed that the survival rate of the 50 mg/kg MC-3129 group were higher than that of the vehicle control group during the 30 days treatment (*P* < 0.05) (Fig. 6c). In contrast, the treatment of nude mice with MC-3129 did not cause significant changes in body weight (Fig. 6d) or other signs of potential toxicity, such as agitation, impaired movement and posture, indigestion, or diarrhea. These results suggest that MC-3129 exhibited more potent inhibitory effects on tumor growth without any acute toxicity.

To clarify whether the inhibition of tumor growth is solely due to the inhibition of cell viability, we investigated the effects of MC-3129 on apoptosis in tumor tissues using H&E staining, TUNEL, and immunohistochemical assays. The sections of U937 xenografts from mice treated with MC-3129 exhibited a reduced number of cancer cells, with signs of necrosis with inflammatory cell infiltration and fibrosis (Fig. 6e, top panels). Exposure to MC-3129 resulted in a striking induction of apoptosis in tumor cells, with signs of numerous dark brown-colored apoptotic cells (Fig. 6e, middle panels). In addition, treatment with MC-3129 caused a rapid increase in immunoreactivity for cleaved caspase-3, which was indicative of apoptosis (Fig. 6e, bottom panels).

To further evaluate whether the interruption of the ROCK1/PTEN/Akt signaling pathway is involved in MC-3129-induced apoptosis in vivo, we performed western blot analyses. As shown in Fig. 6f, the treatment of nude mice with MC-3129 resulted in decreased ROCK1 levels and increased ROCK1 cleavage. Treatment with MC-3129 also increased the levels of phospho-PTEN and decreased the levels of phospho-PI3K, phospho-Akt, and phospho-cofilin in whole cellular lysates of tumor tissues. Such findings suggest that the interruption of the ROCK1/PTEN/PI3K/Akt signaling pathway could contribute to MC-3129-mediated apoptosis and antileukemic effects in vivo.

### Target prediction of MC-3129 by homology modeling and molecular docking

**Fig. 7:**
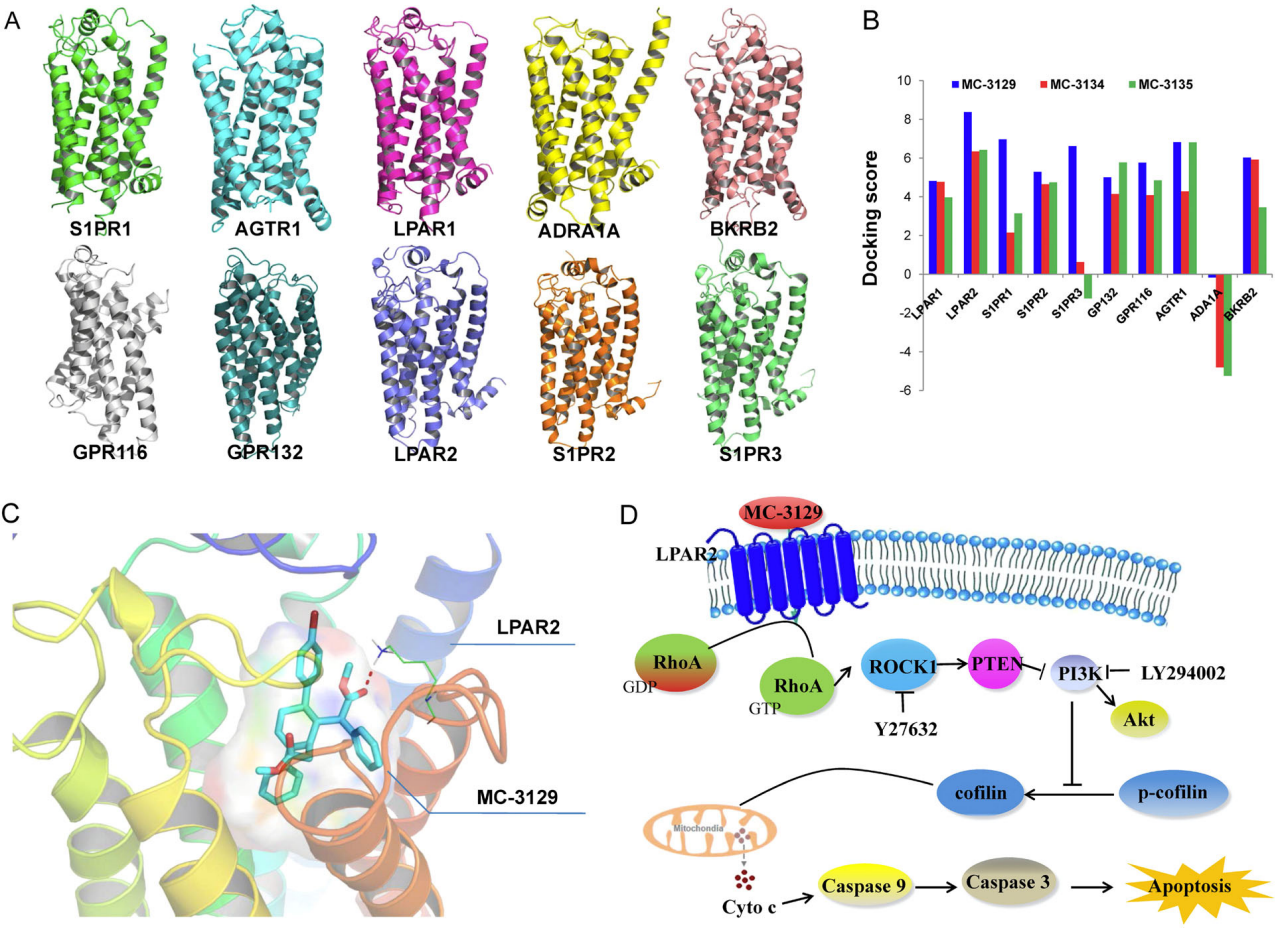
The 3D structures of ten GPCRs that service as upstream effectors of RhoA. **a** The 3D structures of ten GPCRs were generated from RCSB Protein Data Bank (S1PR1 with ID 3VZY, AGTR1 with ID 4YAY, LPAR1 with ID 4Z35) or constructed by homology modeling (for ADRA1A, BKRB2, GPR116, GPR132, LPAR2, S1PR2, and S1PR3). **b** Molecular docking of these GPCRs with compounds (MC-3129, MC-3134, and MC-3135) was performed with SYBYL-X 2.0 Surflex-Dock. The results showed that LPAR2 was the most likely target because the docking scores of compounds with LPAR2 were greater than 6.0. **c** MC-3129 was precisely docked into the binding pocket with an obvious hydrogen bond between LAPR2 and MC-3129. **d** The mechanism of MC-3129 in the regulation of cofilin mitochondrial translocation was predicted as the LPAR2/RhoA/ROCK1/PTEN/PI3K pathway

Ten GPCRs, including lysophosphatidic acid receptor 1, 2 (LPAR1-2), sphingosine 1-phosphate receptor 1, 2, 3 (S1PR1-3), G-protein-coupled receptor 132 (GPR132), G-protein-coupled receptor 116 (GPR116), type-1 angiotensin II receptor (AGTR1), alpha-1A adrenergic receptor (ADRA1A), and B2 bradykinin receptor (BKRB2), were selected as the potential target proteins. The structures of S1PR1, AGTR1, and LPAR1 were obtained from the RCSB Protein Data Bank with ID numbers 3VZY, 4YAY, and 4Z35, respectively (Fig. 7a). Homology modeling was used to construct the remaining GPCRs by using different templates, as shown in Table S1, by Modeler 9.12. Surflex-Dock from SYBYL-X 2.0 was used for docking 12 selected compounds, including both active and inactive compounds (Table S2), into these GPCRs. We selected docking scores greater or equal to 6.0 as the cutoff values, according to SYBLY software, as previously described. The docking results of these compounds with the ten GPCR models are shown in Table S3. Only the LAPR2 model could distinguish active and inactive compounds (Fig. 7b). However, MC-3129 was precisely docked into the binding pocket with an obvious hydrogen bond between LAPR2 and MC-3129 (Fig. 7c). These results suggested that the LPAR2 could be a potential target for these compounds. Therefore, we suggested that MC-3129 might activate the RhoA/ROCK1 pathway by targeting LPAR2 (Fig. 7d).

## Discussion

In this study, we demonstrated that the cyclohexene derivative MC-3129 specifically reduces the viability of human hematological and solid tumor cell lines and exhibits antileukemic activity in vivo without side effects. Such a broad-spectrum anticancer agent with low toxicity is considered promising for the development of anticancer therapies. We also observed that MC-3129 selectively induces apoptosis in leukemia U937 cells in dose- and time-dependent manners. To better characterize this molecule, we investigated the detailed mechanism of MC-3129-induced cell death in U937 cells.

Cofilin is a member of the ADF/cofilin family, which regulates actin dynamics by increasing the rate of actin depolymerization^18^. Cofilin not only serves as an actin-depolymerizing factor, but also plays crucial roles in various cellular activities (i.e., apoptosis)^19^. Recent evidence has indicated that the mitochondrial translocation of cofilin is an early step in apoptosis induction^20^. The translocation of cofilin to mitochondria is necessary for the opening of the mitochondrial permeability transition pore and subsequent release of cytochrome c. Only dephosphorylated cofilin translocated to mitochondria, resulting in cytochrome c release and apoptosis^21^. Consistent with these reports, the dephosphorylation and mitochondrial translocation of cofilin is necessary for MC-3129-mediated cytochrome c release and apoptosis based on the following findings. First, after MC-3129-induced apoptosis, cofilin was translocated from the cytosol to mitochondria prior to the release of cytochrome c. Second, MC-3129 treatment decreased the levels of phosphorylated cofilin. Third, dephosphorylated cofilin enhanced, whereas phosphorylated cofilin attenuated apoptosis mediated by MC-3129. Such findings suggest that the MC-3129-mediated dephosphorylation of cofilin (Ser3) is required for the translocation of cofilin to mitochondria, leading to cytochrome c release and apoptosis induction.

Our results provide detailed information on the molecular mechanisms by which MC-3129 induces apoptosis in human leukemia cells (i.e., by activation of RhoA/ROCK1/PTEN and inactivation of PI3K/Akt). Rho kinase (ROCK) belongs to a family of serine/threonine kinases activated via interactions with Rho GTPases. ROCK is involved in a wide range of fundamental cellular functions, such as contraction, adhesion, migration, proliferation, and apoptosis^22^. The high incidence of overexpression of ROCK1 in human tumors suggests that this kinase is important in the carcinogenic process and therefore may be a potential target for therapeutic intervention. Recent studies have shown that ROCK1 plays an important role in the regulation of apoptosis in various cell types and animal disease models. ROCK1 activity can be regulated by several distinct mechanisms (i.e., RhoA- or caspase-3-dependent cleavage/activation of ROCK1)^23^. We show here that the pan-caspase inhibitor z-VAD-fmk failed to prevent MC-3129-mediated ROCK1 cleavage/activation. However, depletion of RhoA with shRNA could attenuate MC-3129-mediated ROCK1 activation. Such findings suggest that MC-3129-mediated ROCK1 activation is RhoA-dependent. As previously reported, RhoA is a proximal downstream effector of numerous GPCRs^24,25^. Computer modeling revealed that MC-3129 might activate the RhoA/ROCK1 pathway by targeting LPAR2.

Several ROCK substrates are involved in the regulation of cell death and survival^26^. Phosphatase and tensin homolog (PTEN) is a newly identified ROCK substrate^27^, and phosphorylation by ROCK stimulates the phosphatase activity of PTEN. PTEN dephosphorylates both proteins and phosphoinositides and negatively regulates the activities of the phosphatidylinositol (PI) 3-kinase/Akt pathway, which plays important roles in a diverse range of biological processes, including cell survival and apoptosis^28,29^. Based on our results, we speculate that the PTEN/PI3K/Akt signaling cascade acts downstream of the RhoA/ROCK1 pathway during the MC-3129-mediated mitochondrial translocation of cofilin and induction of apoptosis. First, treating U937 cells with MC-3129 induced the activation of RhoA/ROCK1/PTEN and inactivation of PI3K/Akt. Second, pretreatment with ROCK1 inhibitor Y27632 attenuated the MC-3129-mediated activation of PTEN, inactivation of Akt, dephosphorylation, and mitochondrial translocation of cofilin, as well as induction of apoptosis. Third, the knockdown of ROCK1 with siRNA attenuated the MC-3129-mediated activation of PTEN, inactivation of Akt, dephosphorylation, and mitochondrial translocation of cofilin, as well as induction of apoptosis.

In this study, a solid tumor xenograft-like mouse model was employed to evaluate the inhibitory effects on tumor growth of MC-3129 in vivo^30,31^. Our results revealed that MC-3129 inhibited tumor growth in a U937 cell xenograft mouse through the induction of apoptosis (i.e., increased apoptosis and immunoreactivity for cleaved caspase-3). Additionally, the treatment of nude mice with MC-3129 increased the cleavage/activation of ROCK1 and the levels of phospho-PTEN, and decreased the levels of phospho-Akt in tumor sections of nude mice, further confirming the antileukemic effect of MC-3129 through interruption of the ROCK1/PTEN/Akt signaling pathway.

In conclusion, the present findings demonstrated that MC-3129 exerts its selective anticancer effect by inducing cytotoxicity in different types of cancer cell lines. MC-3129 also exhibits its anticancer property by inducing apoptosis in hematological malignancy. Collectively, these findings suggest a hierarchy of events in MC-3129-induced apoptosis, in which RhoA/ROCK1 activation is the primary insult leading to PTEN activation and PI3K/Akt inactivation, resulting in the dephosphorylation and mitochondrial translocation of cofilin, and culminating in cytochrome c release and apoptosis induction (Fig. 7d). Since MC-3129 exhibits broad-spectrum anticancer effects with low toxicity, MC-3129 may have potential for development as a chemotherapeutic agent for the treatment of human leukemia and other cancers. Future preclinical studies should confirm the usefulness of this molecule as a clinical drug candidate for cancer treatment.

## Materials and methods

### Chemicals and antibodies

Y27632 was purchased from Santa Cruz Biotechnology (Santa Cruz, CA). Z-VAD-fmk was purchased from EMD Biosciences (La Jolla, CA). Antibodies against Akt, cytochrome c, cofilin, actin, cleaved ROCK1, PTEN, and GAPDH were obtained from Santa Cruz Biotechnology (Santa Cruz, CA); cleaved caspase-3, cleaved caspase-9, phospho-Akt (Ser473), phosphor-Cofilin (Ser3), PI3K, phospho-PI3K, RhoA, and Cox IV were purchased from Cell Signaling Technology (Beverly, MA); ROCK1 was obtained from Abcam (Burlingame, CA); PARP was obtained from Biomol (Plymouth Meeting, PA).

### Cell culture and establishment of shRNA stable cell line

U937, Jurkat, HL-60, K562, SMMC-7721, and Eca109 cells were cultured in RPMI-1640 medium, while A549, DU145, and MDA-MB-231 cells were cultured in Dulbecco’s modified eagle medium (DMEM); both media contained 10% fetal bovine serum (FBS) and antibiotics. All cell lines were obtained from the American Type Culture Collection (Manassas, VA) and cultured at 37 °C in a humidified atmosphere and 5% CO_2_ in air. The human ROCK1 shRNA (5′-CCGGGCACCAGTTGTACCCGATTTACTCGAGTAAATCGGGTACAACTGGTGCTTTTTG-3′), RhoA shRNA (5′-CCGGCGATGTTATACTGATGTGTTTCTCGAGAAACACATCAGTATAACATCGTTTTTG-3′) were synthesized and subcloned into the pLKO.1 plasmid. At 48 h after the co-transfection of lentiviral packaging plasmids into 293T cells, the lentivirus-containing supernatant was collected. U937 cells were transduced with serial dilutions of lentiviral supernatant in the presence of 5 μg/ml of polybrene and selected by 5 mg/ml of puromycin. After antibiotic selection for 3 weeks, stable shRNA cells were obtained.

### Cell viability (MTT) assay

Approximately 3000 A549, SMMC-7721, Eca109, DU145, MDA-MB-231 cells, and 30,000 U937 cells were seeded onto each well of a 96-well plate. The cells were treated as indicated for 24 h, depending on the experimental conditions. Next, 20 μl of MTT (5 mg/ml) was added per well, and the cells were incubated at 37 °C for 4 h. For the adherent cell lines, the medium was discarded, and the formazan was dissolved in 150 μl of DMSO. The rate of color production was measured at 495 nm by iMark™ Microplate Absorbance Reader (Bio-Rad, Hercules, CA). For suspension cells, 100 μl of 10% SDS, 5% isobutyl alcohol, and 12 mM of HCl were directly added to the medium^32^, and the next day, the plates were read at 595 nm. The cell viabilities were normalized to the control group. The data were subjected to GraphPad Prism 5.01 software (GraphPad Software Inc., San Diego, CA), and the IC_50_ values were calculated by using the respective regression equation according to the best straight-line fit obtained from linear-regression analysis and the regression lines.

### Apoptosis analysis

The extent of apoptosis of the leukemia cells was evaluated by flow cytometry by using the Annexin V/PI Staining Kit (Pharmingen, San Diego, CA) according to the manufacturer’s instructions. Briefly, 1 × 10^6^ cells were washed twice with phosphate-buffered saline (PBS) and stained with 5 μl of Annexin V-FITC and 10 μl of PI (5 μg/ml) in 1× binding buffer (10 mM of HEPES, pH 7.4, 140 mM of NaOH, and 2.5 mM of CaCl_2_) for 15 min at room temperature in the dark. The apoptotic cells were determined by using a Becton-Dickinson FACScan cytofluorometer (Mansfield, MA). Both early (Annexin V-positive, PInegative) and late (Annexin V-positive and PI-positive) apoptotic cells were included in the cell death determinations.

### Preparation of the mitochondrial and cytosolic fractions

Mitochondrial and cytosolic fractions were obtained as previously described^33^. Briefly, cell pellets were washed with cold PBS and resuspended in 5× buffer A (20 mM of HEPES, 10 mM of KCl, 1.5 mM of MgCl_2_, 1 mM of EDTA, 1 mM of EGTA, 1 mM of Na_3_VO_4_, 2 mM of leupeptin, 1 mM of PMSF, 1 mM of DTT, 2 mM of pepstatin A, and 250 mM of sucrose). For homogenization, the cells were passed through a 22-gauge needle 25 times. The homogenate was centrifuged at 4 °C in three sequential steps as follows: 1000 g, 10,000 g, and 100,000 g. The 10,000-g pellet was considered as the “mitochondrial” fraction, and the 100,000 g supernatant was considered as the “cytosolic” fraction. These fractions were subjected to western blot analysis.

### Western blotting

Total cellular samples were washed two times with ice-cold PBS and subsequently lysed in 4× NuPAGE LDS sample buffer (Invitrogen, NP0007) supplemented with 50 mM of dithiothreitol. Protein concentrations were determined using an Enhanced BCA Protein Assay Kit (Beyotime Biotechnology, P0011), and 30 μg of sample proteins was separated using SDS-PAGE and transferred to PVDF membranes (Bio-Rad, 162-0177). The membranes were blocked with 5% fat-free dry milk in Tris-buffered saline (TBS; 10 mM of Tris-Base, 150 mM of NaCl, pH 7.6), containing 0.1% Tween-20 (Santa Cruz Biotechnology, sc-29113) and subsequently incubated with antibodies. The protein bands were detected by incubating with horseradish peroxidase-conjugated secondary antibodies (Kirkegaard and Perry Laboratories, Gaithersburg, MD) and visualized with Clarity Western ECL Substrate (Bio-Rad, 1705061).

### RhoA activity assay

The RhoA activity assays were performed according to the manufacturer’s instructions (Cytoskeleton, Denver, CO). Briefly, 5 × 10^5^ cells were plated and cultured for 2 days. The samples were then rapidly lysed at 4 °C and incubated with sepharose-bound Rhotekin to pull down active RhoA. After washing, the bead/protein complexes were boiled in sample buffer and separated by SDS-PAGE. The blots were incubated with an antibody against RhoA.

### Site-directed mutagenesis and transfection

Dephosphorylated (active, S3A) and pseudophosphorylated (inactive, S3E) human cofilin plasmids were a gift from Professor James Bamburg (Colorado State University, USA). Plasmids were transfected into U937 cells using Lipofectamine 3000 according to the manufacturer’s instructions. After 48 h of transfection, the cells were exposed 10 μM MC-3129 for 24 h and subsequently subjected to immunoblotting or apoptosis analysis.

### Animal studies

Nude mice (5 weeks old) were purchased from Vital River Laboratories (VRL, Beijing, China) and fed a standard animal diet and water. Animal studies were approved by the University Institutional Animal Care and Use Committee. The lower back of each mouse was subcutaneously inoculated with 2 × 10^6^ U937 cells in serum-free RPMI-1640 medium with a Matrigel basement membrane matrix (Sigma, E1270). The mice were randomized into three groups (*n* = 12 for each group). Five days after tumor inoculation, the mice were treated with MC-3129 (10 mg/kg, 50 mg/kg intraperitoneally for 30 days, respectively) or an equal volume of vehicle. The tumor volumes and body weights were monitored every 5 days after treatment. The tumor volumes were determined by measuring tumor length (l) and width (w), and subsequently, the tumor volume was calculated by using *V* = *lw*^2^/2. The mice were killed after 30 days of exposure, and tumor tissues from the representative mice were fixed in paraformaldehyde, embedded in paraffin, sectioned and processed for hematoxylin and eosin (H&E) staining. The TUNEL assay was performed according to the manufacturer’s instructions by using the In Situ Cell Death Detection Kit (Roche, Mannheim, Germany) to detect apoptosis in the tumor tissues. Immunohistochemistry was performed as previously described^34^.

### Preparation of crystal structure of GPCR proteins

Since there are no available crystal structures for ADRA1A, BKRB2, GPR116, GPR132, LPAR2, S1PR2, and S1PR3, some known GPCRs crystal structures with different sequence identities were selected to generate 3D structures of these proteins by using a reported protocol^35^. Their structures were retrieved from the Protein Data Bank (http://www.pdb.org/pdb/) and prepared by SYBYL-X 2.0 (including repair of residues and minimization of energy).

### Homology modeling

The full sequences of ADRA1A, BKRB2, GPR116, GPR132, LPAR2, S1PR2, and S1PR3 were retrieved from the UniProtKB/Swiss-Prot (http://www.uniprot.org/uniprot). In addition to the resolution, the sequence identities generated by sequence alignments of targeted proteins and known crystal structure of GPCRs were used to select appropriate templates. According to Disulfide Bridge from UniProtKB/Swiss-Prot, the disulfide bridges/bonds in the targeted proteins were patched. After aligning the sequences of targeted proteins with the appropriate templates, the alignment was manually adjusted according to the numbers of residues. The conserved motifs in the GPCRs (for example, “D/ERY” in TM3, “CWxPx” or “D/E6.30” in TM6 and “NPxxY” in TM7) were also applied to ensure the reasonability of the TM alignments. These models were constructed by Modeler 9.12^36^. The visualization of the generated models was performed using the PyMOL program.

### Molecular docking

Five compounds with IC_50_ values less than 50 μM were classified as active compounds, while the remaining seven compounds were classified as compounds. Surflex-Dock from SYBYL-X 2.0 was used for docking these compounds to ten GPCR models, in which the total Score was expressed in −log_10_(*K*_d_)^37^. MOLCAD module was implemented in SYBYL-X 2.0 to explore the potential binding pocket for the GPCR models based on the following parameters: minimum dots of 1000, dot density of 6.0 points/area, and probe radius of 1.4 Å. The main protocols or the parameters set for docking included the following criteria: (1) Additional starting conformations per molecule were set to 10. (2) Max number of rotatable bonds per molecule was set to 100. (3) Maximal number poses per molecule was set to 20. (4) Density of search and number of spins per alignment was set to 9.0 and 20, respectively. (5) Pre-dock minimization, post-dock minimization, molecule fragmentation, ring flexibility, and soft grid treatment were turned on in the present work.

### Statistical analysis

Statistical analysis was performed with SPSS 20 software (SPSS, Chicago, Illinois). The data are presented as the means ± SD. For comparison between two data sets, Student’s *t* test was used. For the analysis of three or more sets of data, ANOVA was used. *P *<* 0.05 and **P *<* 0.01 were considered statistically significant.

## Electronic supplementary material

Figure S1

Figure S2

supplementary figure legends

Supplementary Tables
