# Supplementary figures and images for: The cyclohexene derivative MC-3129 exhibits antileukemic activity via RhoA/ROCK1/PTEN/PI3K/Akt pathway-mediated mitochondrial translocation of cofilin
Source: Cell Death Dis. 2018 May 29;9(6):656. doi: 10.1038/s41419-018-0689-4 (PMC5974298; doi:10.1038/s41419-018-0689-4)

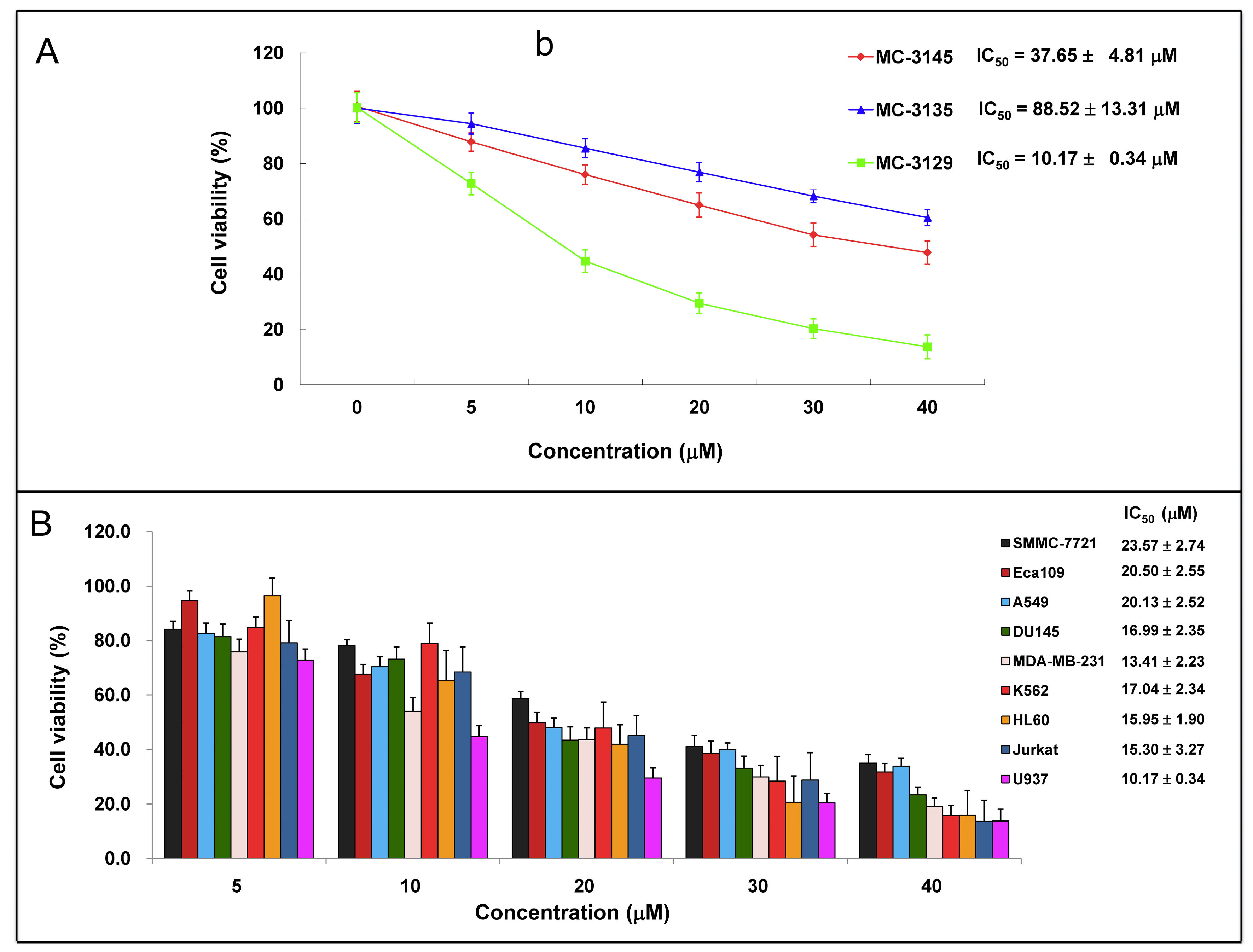

Supplement: Supplementary file 1 — Figure S1 [file 41419_2018_689_MOESM1_ESM.jpg]

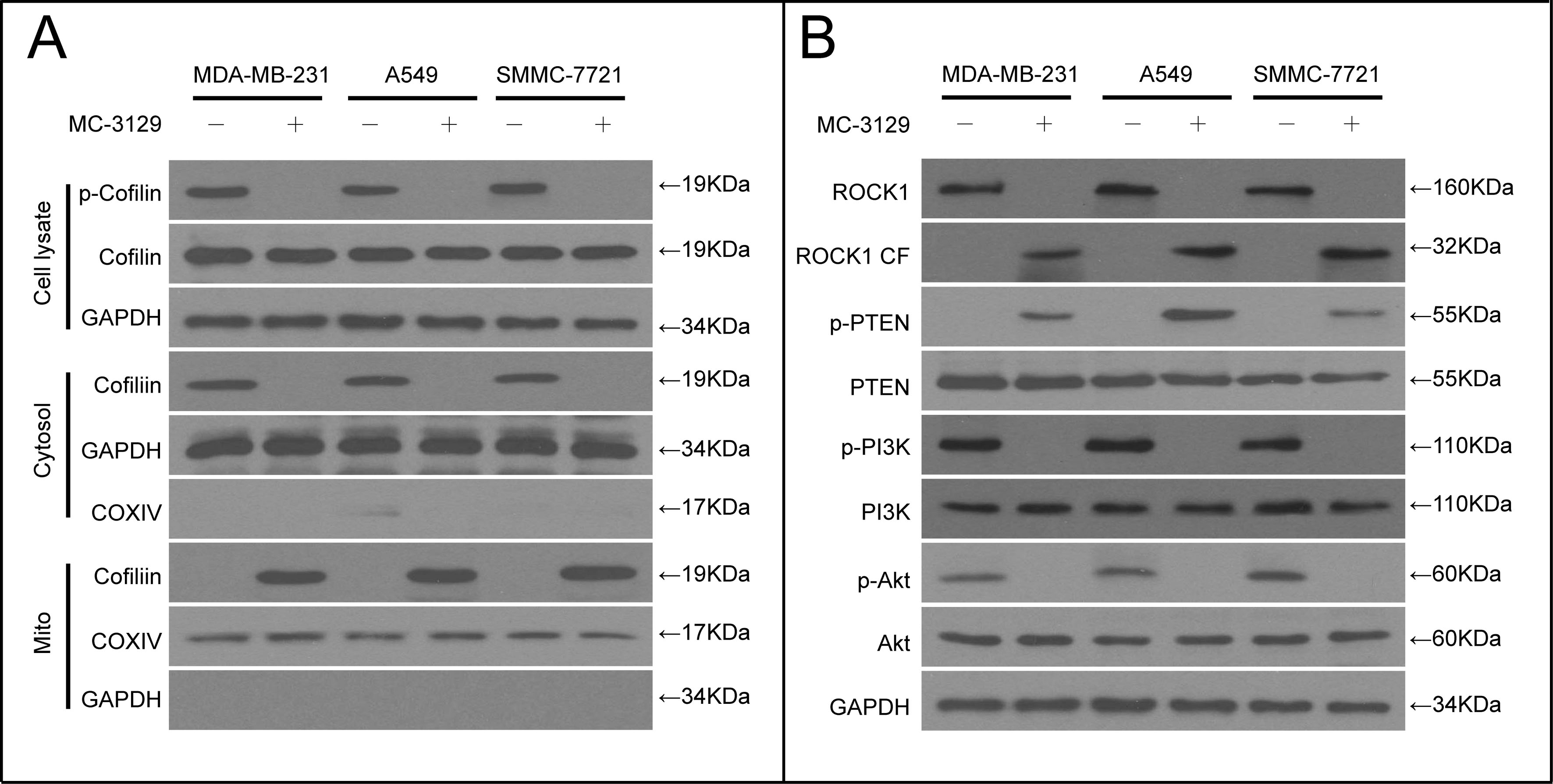

Supplement: Supplementary file 2 — Figure S2 [file 41419_2018_689_MOESM2_ESM.jpg]
