# Supplementary material for: The cyclohexene derivative MC-3129 exhibits antileukemic activity via RhoA/ROCK1/PTEN/PI3K/Akt pathway-mediated mitochondrial translocation of cofilin
Source: Cell Death Dis. 2018 May 29;9(6):656. doi: 10.1038/s41419-018-0689-4 (PMC5974298; doi:10.1038/s41419-018-0689-4)
Supplement: Supplementary file 3 — supplementary figure legends [file 41419_2018_689_MOESM3_ESM.doc]

**The cyclohexene derivative MC-3129 exhibits antileukemic activity via RhoA/ROCK1/PTEN/PI3K/Akt pathway-mediated mitochondrial translocation of cofilin**

Yi Zheng^1^, Qin Ouyang^1^, Ruoqiu Fu, Lei Liu, Hongwei Zhang, Xiaoye Hu, Yanxia Liu, Yingchun Chen^*^, Ning Gao^*^

**Authors’ Affiliations**:

College of Pharmacy, Third Military Medical University, Chongqing 400038, China

^1^These authors contributed equally to this work

^*^**Corresponding author:** Professor. Ning Gao: E-mail: [gaoning59@163.com](mailto:gaoning59@163.com)

Professor. Yingchun Chen: E-mail: [ycchen@scu.edu.cn](mailto:ycchen@scu.edu.cn)

**Running title:** MC-3129 exhibits antileukemic activity

**Word count:** 3264

**Total number of figures:** 9

**Figure S1: MC-3129 inhibits cell viability in multiple cancer cell lines.**

**(A)** U937 cells were treated with increasing doses of MC-3134, MC-3135 and MC-3129 for 24 h, and MTT assays were performed to assess cell viability. IC_50_ values are presented as the means ± SD for three separate experiments. **(B)** Multiple cancer cell lines were treated with increasing doses of MC-3129 for 24 h, and cell viability were measured by MTT assay. IC50 values are presented as the means ± SD for three separate experiments.

**Figure S2：Exposure of MC-3129 affects ROCK1/PTEN/PI3K/Cofilin signaling pathway in diverse cancer cells.**

MDA-MB-231, A549 and SMMC-7721 cells were treated with 10 μM of MC-3129 for 24 h. **(A)** Whole cell lysates, cytosolic (Cytosol) and mitochondrial (Mito) fractions were analyzed by western blot assay using antibodies against phospho-cofilin (p-cofilin), cofilin, GADPH and Cox IV. **(B)** Whole cell lysates were prepared and subjected to western blot analysis using antibodies against against ROCK1, phospho-PTEN (p-PTEN), PTEN, phospho-PI3K (p-PI3K), PI3K, phospho-Akt (Ser473, p-Akt), Akt, and GADPH.
