# Supplementary material for: The cyclohexene derivative MC-3129 exhibits antileukemic activity via RhoA/ROCK1/PTEN/PI3K/Akt pathway-mediated mitochondrial translocation of cofilin
Source: Cell Death Dis. 2018 May 29;9(6):656. doi: 10.1038/s41419-018-0689-4 (PMC5974298; doi:10.1038/s41419-018-0689-4)
Supplement: Supplementary file 4 — Supplementary Tables [file 41419_2018_689_MOESM4_ESM.doc]

**Table 1S.** Information of the crystal structures of GPCRs for homology modeling used in the present work *^a^*.

|  | ADRA1A | BKRB2 | GPR116 | GPR132 | LPAR2 | S1PR2 | S1PR3 |
| --- | --- | --- | --- | --- | --- | --- | --- |
| Template | β1AR | CXCR4 | CRF1R | P2RY1 | LPAR1 | S1PR1 | S1PR1 |
| PDB entry | 4BVN | 4YAY | 4K5Y | 4XNV | 4Z35 | 3V2Y | 3V2Y |
| Resolution (Å) | 2.10 | 2.90 | 2.98 | 2.20 | 2.90 | 2.80 | 2.80 |
| Sequence identity (±2%) | 38.2 | 31.5 | 27.0 | 17.8 | 54.0 | 50.3 | 53.9 |

*^a^*We listed the information of 7 GPCRs used as templates for building homology models, including PDB entry, resolution, sequence identity to targeted receptors.

**Table 2S.** The information of the selected compounds with anti-cancer activity for different cell lines（IC50，μM）


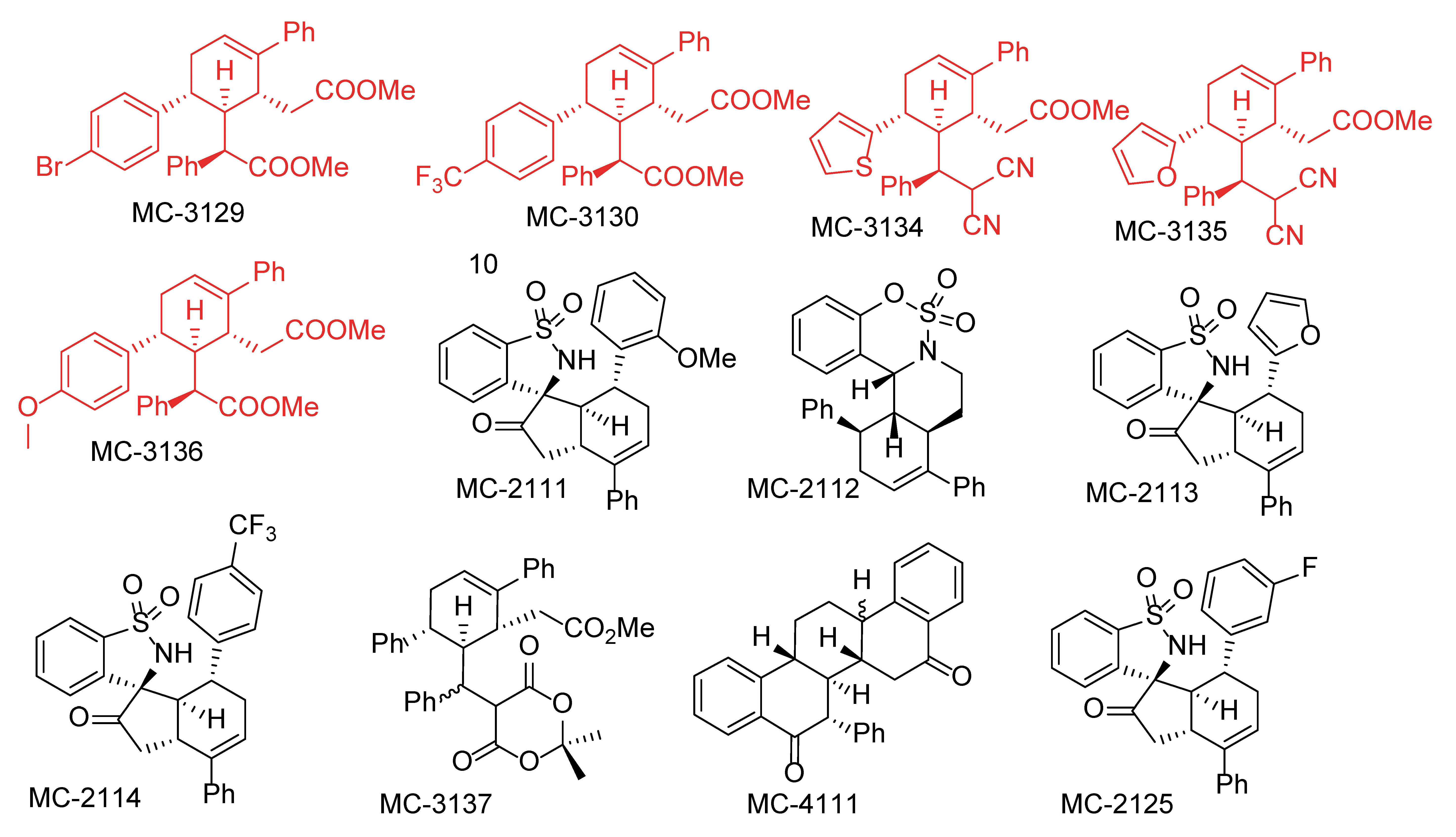


|  | **A549** | **DU145** | **Eca109** | **MDA-MB-231** | **U937** |
| --- | --- | --- | --- | --- | --- |
| **MC-3129** | 30.13 | 16.99 | 20.50 | 13.41 | 10.17 |
| **MC-3130** | 18.82 | 17.14 | 24.6 | 27.24 | 34.41 |
| **MC-3134** | 27.02 | 39.83 | 27.13 | 22.1 | 37.65 |
| **MC-3135** | 34.56 | 29.96 | 36.05 | 26.64 | 88.52 |
| **MC-3136** | 26.52 | 30.84 | 44.85 | 36.62 | 27.15 |
| **MC-2111** | 87.41 | 51.14 | 37.11 | 62.7 | >200 |
| **MC-2112** | 140.4 | >200 | 74.96 | 67.77 | >200 |
| **MC-2113** | >200 | 47.03 | 48.56 | >200 | 61.27 |
| **MC-2114** | 45.11 | >200 | 108.8 | 59.19 | 177.4 |
| **MC-3137** | 71.9 | >200 | 97.79 | 60.92 | 64.96 |
| **MC-4111** | 48.62 | >200 | 90.82 | 58.95 | >200 |
| **MC-2125** | >200 | 38.94 | 47.13 | >200 | 153.2 |

**Table 3S.** The docking scores of 12 selected compounds with 10 GPCRs.

|  | **MC-**  **3129** | **MC-**  **3130** | **MC-**  **3134** | **MC-**  **3135** | **MC-**  **3136** | **MC-**  **2111** | **MC-**  **2112** | **MC-**  **2113** | **MC-**  **2114** | **MC-**  **3137** | **MC-**  **4111** | **MC-**  **2125** |
| --- | --- | --- | --- | --- | --- | --- | --- | --- | --- | --- | --- | --- |
| **LPAR1** | 4.82 | 2.89 | 4.77 | 3.97 | 3.76 | 1.64 | 1.73 | 2.80 | 1.42 | 2.08 | 2.37 | -2.01 |
| **LPAR2** | 8.37 | 7.14 | 6.34 | 6.42 | 7.16 | 4.46 | -0.45 | 2.89 | 3.82 | 1.81 | 4.03 | 1.39 |
| **S1PR1** | 6.96 | 2.06 | 2.15 | 3.14 | -14.57 | 0.76 | -0.36 | -0.64 | -0.23 | 2.10 | 3.20 | 5.22 |
| **S1PR2** | 5.29 | 2.28 | 4.65 | 4.75 | 4.69 | 3.04 | -1.60 | 2.98 | 1.87 | 3.31 | 3.10 | 1.58 |
| **S1PR3** | 6.62 | 0.97 | 0.63 | -1.24 | -3.15 | 6.43 | 4.41 | 4.37 | 2.60 | 1.33 | 1.23 | -13.51 |
| **GPR132** | 5.01 | 7.48 | 4.15 | 5.78 | 5.28 | 3.85 | 5.04 | 3.91 | 4.34 | 6.57 | 5.18 | 7.03 |
| **GPR116** | 5.76 | 5.60 | 4.08 | 4.86 | 5.17 | 4.35 | 5.33 | 3.98 | 3.81 | 5.21 | 3.52 | 3.18 |
| **AGTR1** | 6.83 | 6.72 | 5.28 | 6.81 | 7.79 | 4.53 | 4.25 | 3.75 | 4.40 | 6.67 | 3.88 | 3.30 |
| **ADA1A** | -0.18 | -1.19 | -4.81 | -5.24 | -1.44 | -5.17 | -5.39 | 1.55 | -5.41 | -3.22 | -0.41 | -6.29 |
| **BKRB2** | 6.02 | 6.78 | 5.92 | 3.45 | 8.58 | 5.88 | 2.28 | 3.88 | 5.42 | 7.51 | 4.98 | 5.48 |
